# Perioperative coagulation shifts after degenerative lumbar surgery and exploratory lower-limb thrombosis signals

**DOI:** 10.3389/fsurg.2026.1888316

**Published:** 2026-07-20

**Authors:** Yanjun Zhang, Peng Zhao, Xinwei Zhang, Yadong Liu, Xueming Chen, Liang Liu

**Affiliations:** Department of Bone Center, Beijing Luhe Hospital, Capital Medical University, Beijing, China

**Keywords:** d-dimer, degenerative lumbar disease, fibrinogen, perioperative coagulation, venous thrombosis

## Abstract

**Background:**

Degenerative lumbar disease surgery is associated with perioperative blood loss, tissue injury, postoperative immobility, and activation of hemostatic pathways. Although venous thromboembolism after spine surgery has been investigated extensively, less attention has been given to the integrated perioperative shift of routine hematologic and coagulation markers in degenerative lumbar surgery.

**Methods:**

This single-center retrospective observational study consecutively included patients undergoing surgery for degenerative lumbar disease at Beijing Luhe Hospital, Capital Medical University, between January 1, 2021 and December 31, 2022. After source verification and construction of the locked patient-level analytic cohort, 143 patients were included. Paired preoperative and postoperative day 1 hematologic and coagulation markers were analyzed. Postoperative lower-limb venous thrombosis status was available for 142 patients and was ascertained by clinical records and routine bilateral lower-limb venous ultrasonography, usually performed after drainage tube removal on postoperative day 2 or 3. Thrombosis-stratified comparisons, descriptive ROC analyses, and limited Firth logistic regression were performed only as exploratory and hypothesis-generating analyses.

**Results:**

Ten asymptomatic calf muscular vein thromboses were recorded among 142 patients with available thrombosis status, corresponding to an event rate of 7.0%, and no pulmonary embolism was recorded. Core paired laboratory analyses included 143 preoperative and postoperative day 1 pairs. Hb decreased from 141.00 to 115.00 g/L, HCT from 0.42 to 0.34, and PLT from 216.00 to 201.00 × 10^9/L. APTT shortened from 29.40 to 25.90 s, whereas FIB increased from 2.85 to 3.45 g/L and D-dimer from 0.08 to 0.53 μg/mL DDU; all paired comparisons were statistically significant. In exploratory thrombosis-stratified analyses, patients with ultrasound-detected asymptomatic calf muscular vein thrombosis had higher postoperative D-dimer than those without thrombosis, 1.16 vs. 0.51 μg/mL DDU, lower postoperative APTT, 24.35 vs. 26.00 s, and lower postoperative AT-III, 88.50% vs. 99.00%. In a descriptive ROC analysis, postoperative D-dimer yielded an AUC of 0.715 with bootstrap 95% CI 0.533–0.905.

**Conclusions:**

Routine laboratory markers demonstrated expected perioperative hematologic and coagulation shifts after surgery for degenerative lumbar disease. Exploratory differences in postoperative D-dimer, APTT, and AT-III among patients with ultrasound-detected asymptomatic calf muscular vein thrombosis require prospective validation and are not sufficient for screening, risk stratification, threshold derivation, or prophylaxis decisions.

## Introduction

Degenerative lumbar disease, including lumbar spinal stenosis, lumbar disc herniation, and lumbar spondylolisthesis, is a common cause of pain, disability, neurogenic claudication, and impaired mobility in middle-aged and older adults. Surgical decompression, fusion, and instrumented procedures are frequently performed when conservative treatment fails or neurological impairment progresses (1, 2). Although surgery may improve symptoms and function, it also involves perioperative physiological stress, tissue injury, blood loss, postoperative immobilization, and changes in hemostatic balance.

Venous thromboembolism, including deep vein thrombosis and pulmonary embolism, is an important postoperative complication after spine surgery. The reported incidence varies substantially across studies because of differences in patient population, operative procedure, thrombosis definition, screening strategy, follow-up duration, and prophylaxis protocol (3–5). A systematic review of elective spinal surgery reported an overall VTE incidence of approximately 3.2%, while emphasizing heterogeneity in thromboprophylaxis strategies and outcome ascertainment (5).

Prevention of VTE after spine surgery requires a careful balance between thrombotic risk and bleeding risk. Unlike some major orthopedic procedures, spine surgery carries specific concern regarding postoperative spinal epidural hematoma and neurological compromise. The American Society of Hematology guidelines for surgical hospitalized patients emphasize context-specific prophylaxis decisions based on the balance between VTE risk and bleeding risk (6). Spine-specific antithrombotic guidance also emphasizes individualized decision-making in adult spine surgery (7).

Routine perioperative laboratory markers may reflect different components of the surgical hemostatic response. Hemoglobin and hematocrit reflect perioperative blood loss, hemodilution, and anemia. Platelet count may be influenced by consumption, dilution, inflammation, and marrow response. Fibrinogen is both a coagulation factor and an acute-phase reactant, and postoperative elevation may reflect inflammatory and procoagulant activation. D-dimer is a fibrin degradation product and a marker of coagulation and fibrinolytic activation, although postoperative D-dimer is nonspecific and influenced by surgical trauma, bleeding, inflammation, age, assay type, and sampling time (8, 9).

The main contribution of the present study is not the identification of a new diagnostic or predictive marker, but the integrated description of routine hematologic and coagulation shifts after degenerative lumbar surgery under a real-world institutional perioperative workflow.

Most available spine surgery studies have focused on VTE incidence, risk factors, or prophylaxis. Comparatively fewer studies have evaluated the overall perioperative hematologic and coagulation profile as the primary analytical framework, while treating postoperative thrombosis as an exploratory clinical outcome (10–12). This distinction is important because low event counts often limit the reliability of conventional multivariable modeling and prediction model development (13–17).

Therefore, this single-center retrospective observational study aimed to evaluate perioperative hematologic and coagulation changes in patients undergoing surgery for degenerative lumbar disease. A secondary objective was to explore whether selected postoperative and perioperative-change markers were associated with postoperative lower-limb venous thrombosis.

## Methods

### Study design and setting

This was a single-center retrospective observational study with exploratory thrombosis-related analyses. The study consecutively included patients who underwent surgery for degenerative lumbar disease at Beijing Luhe Hospital, Capital Medical University, from January 1, 2021 to December 31, 2022. Reporting was planned with reference to the STROBE statement (18, 19). The study was approved by the Ethics Committee of Beijing Luhe Hospital, Capital Medical University. The requirement for written informed consent was waived by the Ethics Committee because this retrospective study used deidentified routinely collected clinical data, involved no direct patient contact or additional intervention, and posed minimal risk to participants.

### Participants

Patients were consecutively screened during the study period. The original dataset contained 150 clinical records. Of these, 7 were excluded before construction of the locked patient-level cohort because of incomplete key data required for patient-level verification or paired perioperative analysis. The final locked cohort included 143 unique patients. Patients were included if they underwent surgery for degenerative lumbar disease and had paired preoperative and postoperative day 1 laboratory data available.

### Data source and deidentification

Data were extracted from Excel-based clinical datasets containing perioperative demographic, diagnostic, procedural, laboratory, surgical-segment, prophylaxis-workflow, transfusion-related, and complication-related variables. Direct identifiers, including medical record number, patient name, and free-text remarks, were removed before analysis.

### Variables and laboratory measurements

The main laboratory variables included Hb, HCT, PLT, PT, INR, APTT, TT, FIB, AT-III, and D-dimer measured preoperatively and on postoperative day 1. Absolute change was calculated as postoperative value minus preoperative value. D-dimer was reported in μg/mL DDU according to the institutional laboratory reporting convention, with a reference interval of 0–0.243 μg/mL DDU in the laboratory information system. The exact assay platform used during the study period was not available in the deidentified analytic dataset. Pharmacological anticoagulation was generally not started before postoperative day 1 blood sampling. The log-transformed D-dimer variable was calculated as log(D-dimer+0.01) to reduce skewness and handle near-zero values.

### Surgical and transfusion-related variables

Operated segment information was standardized according to the full marked segment range in the source record. For example, L3–S1 was coded as involving L3/4, L4/5, and L5/S1. Surgical segment information was available for all 143 patients in the locked patient-level cohort and for all 142 patients with available postoperative thrombosis status. For procedures involving two or more operated levels, autologous blood salvage was routinely used according to institutional practice. Allogeneic transfusion was added only in a small number of patients with substantial postoperative blood loss or clinically indicated anemia. Therefore, the transfusion-related variable was interpreted as an autologous blood salvage or transfusion-related record rather than allogeneic red blood cell transfusion alone. Detailed procedure categories, including decompression alone vs. fusion, instrumentation, interbody fusion type, open vs. minimally invasive approach, and revision status, were not reliably available in the structured analytic dataset. Therefore, surgical heterogeneity was described mainly by operated motion segments, operated level distribution, operative time, and intraoperative blood loss.

### Postoperative lower-limb venous thrombosis ascertainment

Postoperative lower-limb venous thrombosis was ascertained from clinical records and routine bilateral lower-limb venous ultrasonography. In the institutional perioperative workflow, bilateral lower-limb venous ultrasonography was routinely performed after drainage tube removal, usually on postoperative day 2 or 3. Among the 142 patients with available thrombosis status, 10 postoperative thrombotic events were recorded. All 10 events were asymptomatic calf muscular vein thromboses detected by routine ultrasonography. No proximal deep venous thrombosis or pulmonary embolism was recorded during complication review. Systematic screening for asymptomatic pulmonary embolism was not performed.

### Institutional VTE prevention workflow

The institutional routine perioperative VTE prevention protocol included in-bed ankle-pump exercises, mobilization when clinically appropriate, postoperative AV pump or intermittent pneumatic compression, and elastic stockings. Drainage tubes were usually removed on postoperative day 2 or 3, after which routine lower-limb venous ultrasonography was ordered and ambulation was permitted when ultrasonography showed no thrombosis-related abnormality. Routine pharmacological anticoagulation was not administered during the early postoperative period. Anticoagulation was added only when postoperative thrombosis was detected, according to clinical evaluation and institutional practice. Accordingly, pharmacological anticoagulation was generally not started before postoperative day 1 blood sampling.

### Outcomes

The primary analytical focus was perioperative hematologic and coagulation change, evaluated by paired comparison of preoperative and postoperative day 1 laboratory markers. The secondary exploratory outcome was postoperative lower-limb venous thrombosis. ROC and Firth logistic regression analyses were performed only to describe exploratory signals and were not intended for threshold derivation, risk stratification, clinical decision-making, or prediction model development.

### Statistical analysis

Continuous variables were summarized as median and interquartile range. Categorical variables were summarized as number and percentage. Paired preoperative and postoperative markers were compared using the Wilcoxon signed-rank test. Between-group comparisons according to thrombosis status were exploratory and used the Mann–Whitney U test for continuous variables and Fisher exact test for categorical variables. No multiplicity correction was applied. ROC analyses were performed for selected markers, with bootstrap 95% confidence intervals for AUC estimates; these analyses were descriptive and were not intended to identify diagnostic thresholds. Given the small number of thrombotic events, Firth penalized logistic regression was used only for limited exploratory association analyses to reduce small-sample bias and separation-related instability (16, 17). Statistical analyses were performed using Python 3.11. Data management was performed using pandas, numerical computations using NumPy, statistical analyses using SciPy and statsmodels, exploratory receiver operating characteristic analyses using scikit-learn, and graphical visualization using Matplotlib. Bootstrap confidence intervals were generated using Python-based resampling procedures.

## Results

### Study population and basic information

Patients were consecutively screened between January 1, 2021 and December 31, 2022. The original dataset contained 150 clinical records. Of these, 7 were excluded before construction of the locked patient-level cohort because of incomplete key data required for patient-level verification or paired perioperative analysis. The final locked cohort included 143 unique patients. Postoperative lower-limb venous thrombosis status was available for 142 patients. Ten asymptomatic calf muscular vein thromboses were detected by routine bilateral lower-limb venous ultrasonography, corresponding to an event rate of 7.0%. No proximal deep venous thrombosis or pulmonary embolism was recorded.

**Figure 1:**
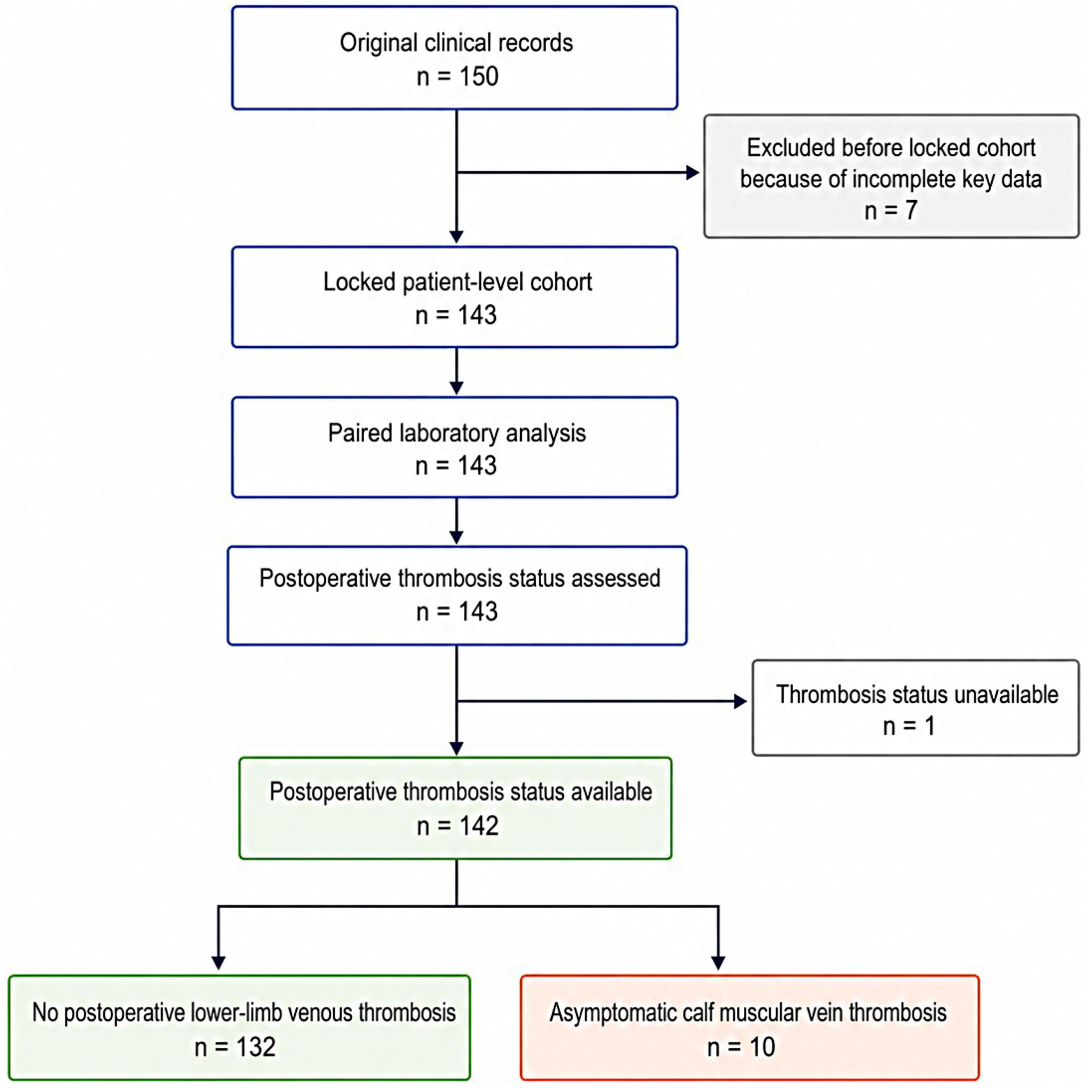
Patient selection, exclusion before construction of the locked cohort, and postoperative thrombosis status.

The thrombosis-available cohort included 109 patients with lumbar spinal stenosis, 28 with lumbar disc herniation, and 5 with lumbar spondylolisthesis. The median age was 65.00 years, and 79 patients were female. Surgical segment information was available for all 142 patients with postoperative thrombosis status. The median number of operated motion segments was 2. Forty-five patients underwent single-level surgery and 97 underwent multilevel surgery. L4/5 was the most frequently involved level. The distribution of operated segments and surgical extent is shown in Table 1 and Figure 1.

**Table 1:** Baseline, surgical extent, and perioperative characteristics according to postoperative lower-limb venous thrombosis.

Characteristic	No thrombosis *n* = 132	Thrombosis *n* = 10	*P* value
Age, years	64.50 [56.75, 70.00]	68.00 [64.75, 72.75]	0.103
Female sex	74 [56.1]	5 [50.0]	0.751
Lumbar spinal stenosis	101 [76.5]	8 [80.0]	1.000
Lumbar disc herniation	27 [20.5]	1 [10.0]	0.687
Lumbar spondylolisthesis	4 [3.0]	1 [10.0]	0.310
Operation time, h	2.50 [2.23, 3.17]	2.70 [2.32, 3.20]	0.629
Intraoperative blood loss, mL	300.00 [200.00, 500.00]	550.00 [262.50, 600.00]	0.131
Number of operated motion segments	2.00 [1.00, 3.00]	2.00 [2.00, 3.00]	0.076
Single-level surgery	45 [34.1]	0 [0.0]	0.031
Multilevel surgery	87 [65.9]	10 [100.0]	0.031
Two-level surgery	52 [39.4]	6 [60.0]	0.317
Three-or-more-level surgery	35 [26.5]	4 [40.0]	0.462
L3/4 involvement	60 [45.5]	9 [90.0]	0.008
L4/5 involvement	113 [85.6]	10 [100.0]	0.358
L5/S1 involvement	70 [53.0]	4 [40.0]	0.520
Thoracolumbosacral long-segment surgery	1 [0.8]	0 [0.0]	1.000
Autologous blood salvage or transfusion-related record	94 [71.2]	9 [90.0]	0.285
ICU admission	2 [1.5]	0 [0.0]	1.000
Postoperative infection	1 [0.8]	0 [0.0]	1.000
Hypertension	61 [46.2]	8 [80.0]	0.051
Diabetes	29 [22.0]	3 [30.0]	0.694
Coronary heart disease history	15 [11.4]	3 [30.0]	0.116
Cerebrovascular disease history	12 [9.1]	0 [0.0]	1.000

Values are median [IQR] or *n* [%]. Between-group comparisons were exploratory and unadjusted.

All thrombosis events occurred among patients undergoing multilevel surgery, and L3/4 involvement was more frequent in the thrombosis group; however, these findings were based on only 10 events, were not adjusted for confounding, and were interpreted only as exploratory descriptive findings.

### Paired perioperative hematologic and coagulation changes

**Figure 2:**
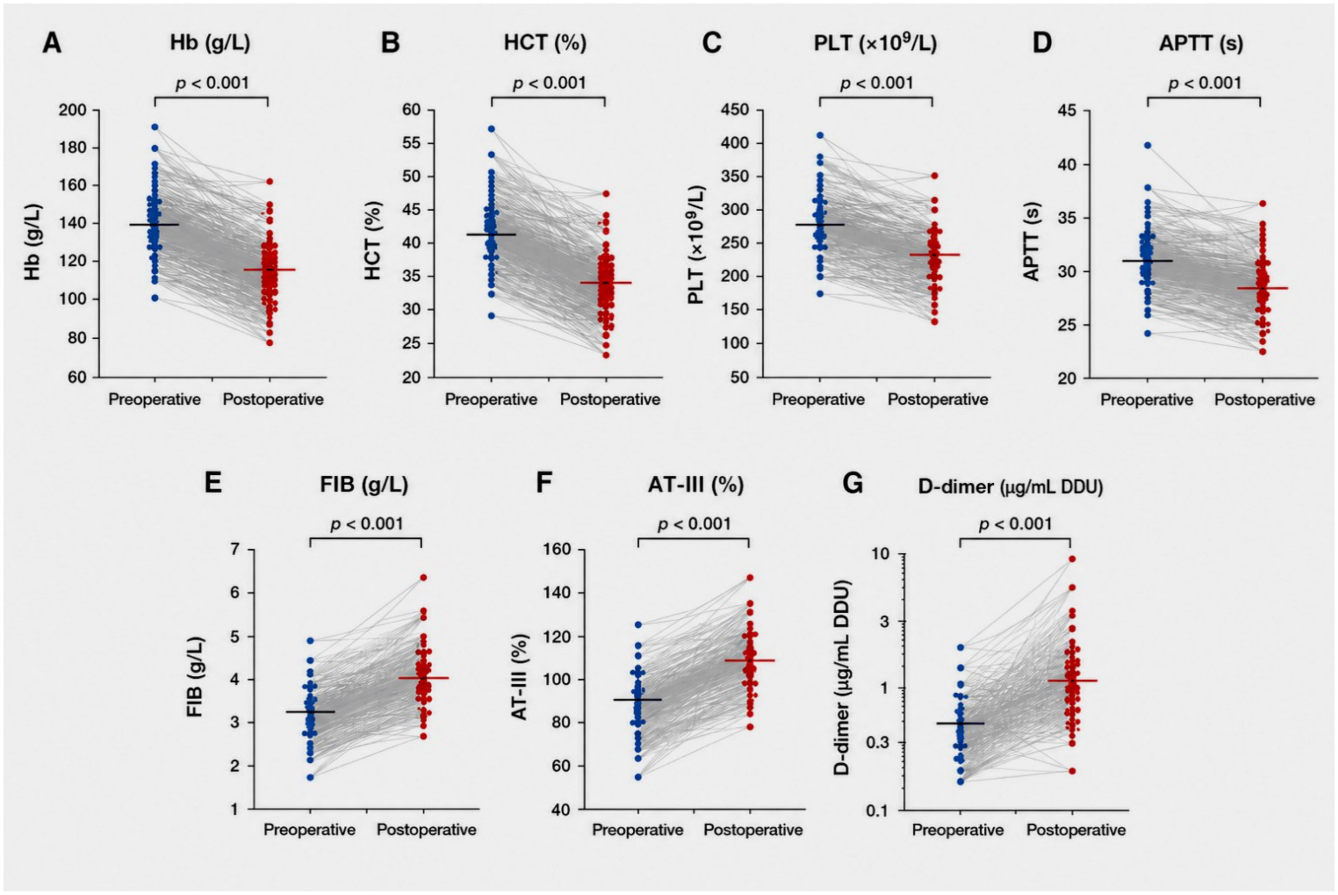
Paired perioperative changes in selected hematologic and coagulation markers. Paired preoperative and postoperative day 1 values are shown for selected hematologic and coagulation markers. Postoperative values showed decreases in Hb, HCT, PLT, and APTT and increases in FIB, AT-III, and D-dimer. Paired comparisons were performed using the Wilcoxon signed-rank test. All displayed comparisons were statistically significant at *p* < 0.001.

Substantial perioperative changes were observed across hematologic and coagulation markers. Hb decreased from 141.00 to 115.00 g/L, HCT from 0.42 to 0.34, and PLT from 216.00 to 201.00 × 10^9/L. APTT shortened from 29.40 to 25.90 s. FIB increased from 2.85 to 3.45 g/L, AT-III from 91.00% to 98.00%, and D-dimer from 0.08 to 0.53 μg/mL DDU. All displayed paired comparisons were statistically significant at *p* < 0.001 (Figure 2, Table 2).

**Table 2:** Paired perioperative hematologic and coagulation changes.

Variable	Preoperative value	Postoperative value	Absolute change	*P* value
Hb, g/L	141.00 [131.00, 153.00]	115.00 [105.50, 127.50]	−25.00 [−31.00, −19.50]	<0.001
HCT, L/L	0.42 [0.39, 0.45]	0.34 [0.30, 0.37]	−0.08 [−0.11, −0.06]	<0.001
PLT, × 10^9/L	216.00 [191.50, 250.00]	201.00 [165.50, 235.50]	−24.00 [−44.00, 0.50]	<0.001
PT, s	10.90 [10.50, 11.60]	11.30 [10.90, 11.80]	0.20 [−0.20, 0.80]	<0.001
INR	0.99 [0.95, 1.05]	1.02 [0.97, 1.07]	0.02 [−0.02, 0.07]	<0.001
APTT, s	29.40 [28.10, 31.40]	25.90 [24.80, 27.95]	−3.60 [−4.95, −2.30]	<0.001
TT, s	15.20 [14.50, 16.05]	14.30 [13.20, 15.50]	−0.90 [−1.75, 0.20]	<0.001
FIB, g/L	2.85 [2.58, 3.14]	3.45 [2.99, 4.29]	0.65 [0.07, 1.46]	<0.001
AT-III, %	91.00 [84.00, 99.00]	98.00 [88.50, 107.50]	6.00 [−2.00, 14.00]	<0.001
D-dimer, μg/mL DDU	0.08 [0.06, 0.13]	0.53 [0.32, 1.03]	0.44 [0.24, 0.85]	<0.001
log(D-dimer + 0.01)	−2.39 [−2.70, −1.96]	−0.61 [−1.11, 0.04]	1.87 [1.32, 2.35]	<0.001

Values are median [IQR]. Absolute change=postoperative day 1 value minus preoperative value. Paired comparisons used the Wilcoxon signed-rank test. D-dimer was reported in μg/mL DDU. The log-transformed variable was calculated as log(D-dimer+0.01).

### Exploratory thrombosis-stratified comparisons

**Figure 3:**
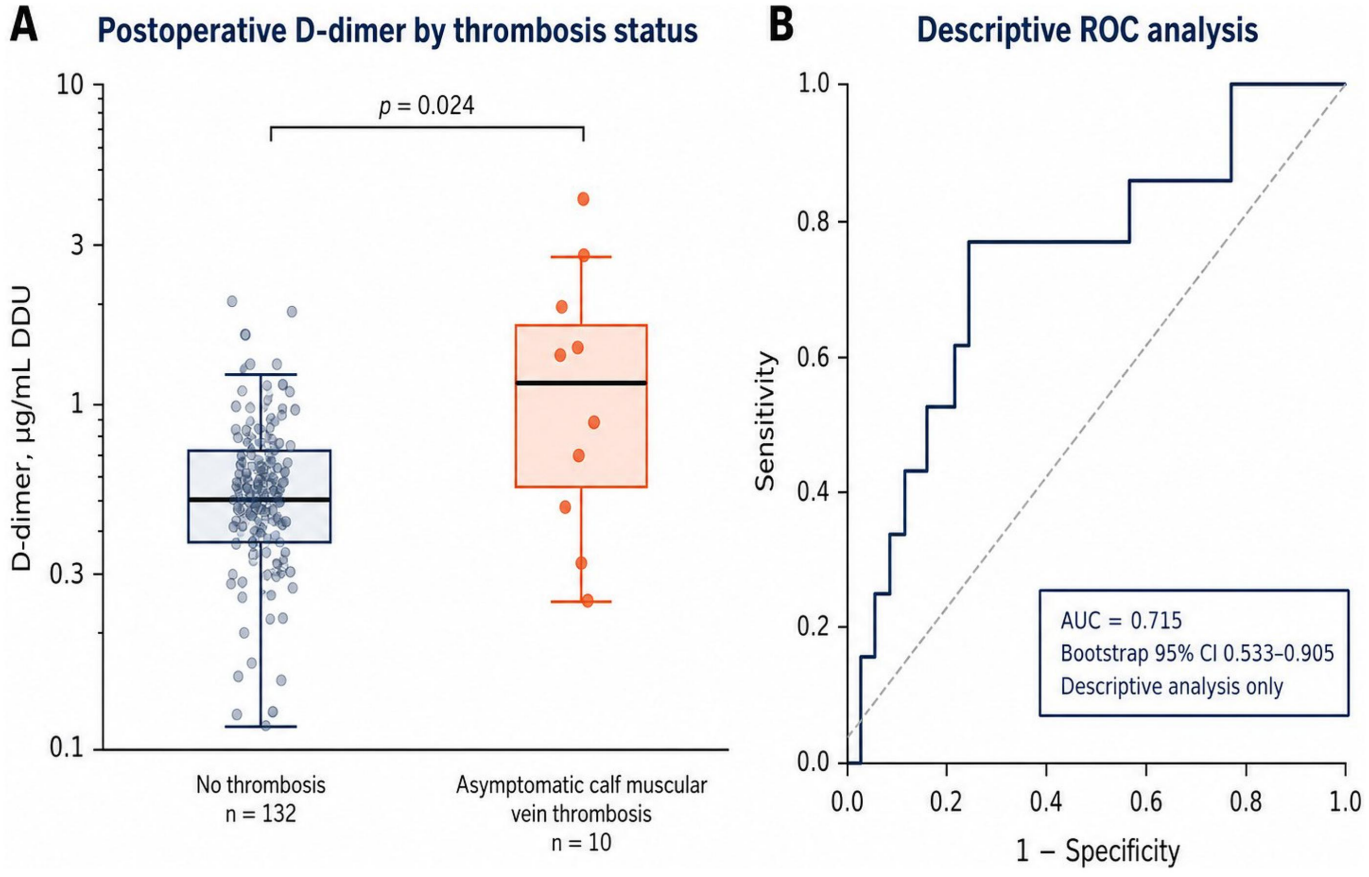
Postoperative D-dimer distribution and descriptive ROC analysis for postoperative lower-limb venous thrombosis. Postoperative D-dimer distributions are shown according to postoperative lower-limb venous thrombosis status. D-dimer was reported in μg/mL DDU. The ROC curve represents a descriptive exploratory analysis of postoperative D-dimer and was not used for threshold derivation or clinical decision-making. The AUC was 0.715 with bootstrap 95% CI 0.533–0.905.

In exploratory unadjusted comparisons, patients with ultrasound-detected asymptomatic calf muscular vein thrombosis had higher postoperative D-dimer than those without thrombosis, 1.16 vs. 0.51 μg/mL DDU. The thrombosis group also showed lower postoperative APTT, 24.35 vs. 26.00 s, and lower postoperative AT-III, 88.50% vs. 99.00%. Because all thrombosis events were asymptomatic calf muscular vein thromboses detected by routine bilateral lower-limb venous ultrasonography, these results should not be generalized to symptomatic VTE, proximal DVT, or pulmonary embolism populations (Figure 3, Table 3).

**Table 3:** Exploratory unadjusted comparison of postoperative coagulation-related markers according to postoperative lower-limb venous thrombosis status.

Variable	No thrombosis *n* = 132	Thrombosis *n* = 10	*P* value
Postoperative D-dimer, μg/mL DDU	0.51 [0.31, 0.93]	1.16 [0.62, 1.99]	0.024
ΔD-dimer, μg/mL DDU	0.42 [0.24, 0.81]	0.95 [0.42, 1.65]	0.090
Δlog(D-dimer + 0.01)	1.86 [1.34, 2.28]	2.17 [0.85, 2.66]	0.670
Postoperative FIB, g/L	3.46 [3.03, 4.29]	3.13 [2.94, 4.11]	0.582
ΔFIB, g/L	0.66 [0.09, 1.45]	0.16 [−0.51, 1.61]	0.376
Postoperative APTT, s	26.00 [24.90, 28.02]	24.35 [23.15, 25.05]	0.037
ΔAPTT, s	−3.65 [−5.00, −2.30]	−3.80 [−4.73, −2.25]	0.886
Postoperative AT-III, %	99.00 [89.68, 108.00]	88.50 [79.50, 98.75]	0.040
ΔAT-III, %	6.50 [−2.00, 14.25]	0.50 [−6.25, 4.75]	0.077
ΔHb, g/L	−25.50 [−31.00, −20.75]	−22.50 [−27.50, −15.75]	0.305

Values are median [IQR]. Comparisons were exploratory and unadjusted. No multiplicity correction was applied. D-dimer was reported in μg/mL DDU.

### Exploratory ROC and Firth logistic regression analyses

In a descriptive ROC analysis, postoperative D-dimer yielded an AUC of 0.715, with bootstrap 95% CI 0.533–0.905. In exploratory Firth logistic regression, postoperative D-dimer showed an exploratory association signal, with an OR of 1.826 per 1 SD increase and 95% CI 1.143–2.916. In a minimal age-adjusted model, the corresponding OR was 1.697, with 95% CI 1.058–2.723. These analyses were descriptive and exploratory and were not intended for threshold derivation, prediction, risk stratification, screening decisions, prophylaxis decisions, or clinical decision-making (Figure 3, Table 4).

**Table 4:** Descriptive ROC and exploratory Firth logistic regression analyses for postoperative lower-limb venous thrombosis.

Analysis	Marker or model	Estimate	95% CI
ROC	Postoperative D-dimer, μg/mL DDU	AUC 0.715	0.533–0.905
ROC	ΔD-dimer, μg/mL DDU	AUC 0.661	0.512–0.844
ROC	Postoperative AT-III	AUC 0.695	0.531–0.849
ROC	Postoperative APTT	AUC 0.698	0.521–0.887
Firth logistic	Postoperative D-dimer per 1 SD	OR 1.826	1.143–2.916
Firth logistic	Postoperative D-dimer per 1 SD in age-adjusted model	OR 1.697	1.058–2.723

ROC analyses were descriptive and exploratory and were not intended for threshold derivation, prediction, risk stratification, screening decisions, prophylaxis decisions, or clinical decision-making. Firth logistic regression was used to reduce small-sample bias and separation-related instability in low-event exploratory analyses.

## Discussion

In this single-center retrospective observational study of patients undergoing surgery for degenerative lumbar disease, we observed substantial perioperative changes in hematologic and coagulation markers. After construction of the locked patient-level cohort, 143 patients were included, and postoperative lower-limb venous thrombosis status was available in 142 patients. Ten asymptomatic calf muscular vein thromboses were detected by routine bilateral lower-limb venous ultrasonography. The principal finding was a consistent perioperative shift characterized by postoperative decreases in Hb, HCT, and PLT, together with shortened APTT, increased FIB, and increased D-dimer. In exploratory thrombosis-stratified analyses, patients with ultrasound-detected asymptomatic calf muscular vein thrombosis had higher postoperative D-dimer, lower postoperative APTT, and lower postoperative AT-III than those without thrombosis. Because only 10 thrombotic events were observed, all thrombosis-related analyses should be interpreted as hypothesis-generating rather than confirmatory.

The main contribution of this study is not the establishment of a predictive biomarker, but the integrated description of routine hematologic and coagulation shifts after degenerative lumbar surgery under a real-world institutional perioperative workflow.

The postoperative decreases in Hb and HCT are consistent with expected perioperative blood loss, hemodilution, and redistribution after lumbar spine surgery. Even without overt hemorrhagic complications, cancellous bone bleeding, epidural venous plexus bleeding, soft-tissue dissection, drainage, and fluid administration can produce measurable changes in red-cell-related markers. The modest platelet decrease may reflect hemodilution, perioperative platelet consumption, inflammatory response, or redistribution.

Fibrinogen increased significantly after surgery, which is biologically plausible because fibrinogen is both a coagulation factor and an acute-phase reactant. D-dimer also increased markedly after surgery. D-dimer reflects fibrin turnover and fibrinolytic activation but is nonspecific after surgery because it may rise with tissue injury, inflammation, wound healing, hematoma resorption, and sampling time (8, 9). Therefore, the observed postoperative D-dimer increase should be interpreted primarily as a marker of perioperative coagulation and fibrinolytic activation rather than direct evidence of thrombosis.

All thrombotic events in this cohort were asymptomatic calf muscular vein thromboses detected by routine bilateral lower-limb venous ultrasonography. Therefore, the 7.0% event rate should not be compared directly with studies limited to symptomatic proximal DVT, clinically diagnosed VTE, or pulmonary embolism. No proximal deep venous thrombosis or pulmonary embolism was recorded, and systematic screening for asymptomatic pulmonary embolism was not performed. These features narrow the interpretation of the thrombosis outcome to ultrasound-detected asymptomatic calf muscular vein thrombosis rather than the full clinical spectrum of VTE.

The institutional workflow emphasized mechanical prevention, ankle-pump exercises, elastic stockings, AV pump or intermittent pneumatic compression, and mobilization after routine ultrasound clearance. Routine pharmacological anticoagulation was not used before postoperative day 1 laboratory sampling, which reduces but does not eliminate the possibility that prophylaxis practices influenced later thrombosis occurrence and coagulation markers.

Although all thrombosis events occurred in multilevel procedures, this finding should not be interpreted as evidence that multilevel surgery independently increases thrombosis risk because the number of events was very small and adjustment for surgical invasiveness, fusion, blood loss, comorbidities, and prophylaxis was not feasible. Similarly, the L3/4 involvement finding should be viewed only as a sparse-data descriptive result rather than a robust association.

The event count was only 10. Therefore, conventional multivariable logistic regression, LASSO, machine learning, nomogram construction, and risk score development was not appropriate. ROC analysis was used only descriptively and should not be used to define a diagnostic threshold. Firth logistic regression was used only to reduce small-sample bias and separation-related instability in low-event exploratory association analyses (16, 17).

These findings describe how routine perioperative laboratory markers changed after lumbar surgery and may help characterize the postoperative hemostatic response at the cohort level. However, they should not be used to guide screening decisions, risk stratification, threshold derivation, prophylaxis decisions, or anticoagulation decisions. VTE prevention guidance emphasizes individualized assessment of thrombotic and bleeding risks (6, 7, 20). Postoperative D-dimer or APTT changes should therefore be interpreted together with clinical symptoms, ultrasound findings, surgical complexity, bleeding risk, mobility status, and institutional prophylaxis protocols.

A recent arthroplasty study also highlighted the importance of distinguishing asymptomatic muscular calf vein thrombosis from axial deep calf vein or proximal deep venous thrombosis. In that study, screen-detected cases were limited to the gastrocnemius or soleal veins and were interpreted within a standardized thromboprophylaxis pathway (21). Although conducted in a different surgical setting, this perspective supports our approach of separating ultrasound-detected calf muscular vein thrombosis from proximal or clinically evident VTE and interpreting these asymptomatic findings with caution.

This study was single-center and retrospective, and is subject to selection bias, information bias, and residual confounding. Only 10 thrombotic events were observed, limiting statistical modeling. All thrombotic events were asymptomatic calf muscular vein thromboses detected by routine bilateral lower-limb venous ultrasonography; therefore, the findings may not be comparable with studies focusing on symptomatic VTE, proximal DVT, or pulmonary embolism. No pulmonary embolism occurred, but systematic screening for asymptomatic pulmonary embolism was not performed.

Detailed patient-level adherence to mechanical prophylaxis, exact mobilization timing, drainage volume, and anticoagulation timing after thrombosis detection were not available. Information on preoperative antiplatelet or anticoagulant management and exact blood sampling clock time was also incomplete. Transfusion-related records included routine autologous blood salvage in multilevel procedures and only a small number of allogeneic transfusions, so this variable should be interpreted cautiously. The multilevel surgery and L3/4 findings are vulnerable to sparse-data bias and multiple testing. These limitations further support interpreting the thrombosis-related findings as exploratory.

## Conclusions

Routine laboratory markers demonstrated expected perioperative hematologic and coagulation shifts after surgery for degenerative lumbar disease. Exploratory differences in postoperative D-dimer, APTT, and AT-III among patients with ultrasound-detected asymptomatic calf muscular vein thrombosis require prospective validation and are not sufficient for screening, risk stratification, threshold derivation, or prophylaxis decisions.

## Data Availability

The original contributions presented in the study are included in the article/Supplementary Material, further inquiries can be directed to the corresponding authors.
